# Discovering a Reliable Heat-Shock Factor-1 Inhibitor to Treat Human Cancers: *Potential Opportunity for Phytochemists*

**DOI:** 10.3389/fonc.2018.00097

**Published:** 2018-04-06

**Authors:** Murugesan Velayutham, Arturo J. Cardounel, Zhenguo Liu, Govindasamy Ilangovan

**Affiliations:** ^1^Center for Biomedical EPR Spectroscopy and Imaging, The Ohio State University, Columbus, OH, United States; ^2^Department of Anesthesiology, Wexner Medical Center, The Ohio State University, Columbus, OH, United States; ^3^Division of Cardiovascular Medicine, Department of Internal Medicine, Davis Heart and Lung Research Institute, The Ohio State University, Columbus, OH, United States

**Keywords:** heat-shock factors, inhibitor, natural products, heat-shock proteins, triptolide, quercetin, pfithrin alpha, cancer

## Abstract

Heat-shock factor-1 (HSF-1) is an important transcription factor that regulates pathogenesis of many human diseases through its extensive transcriptional regulation. Especially, it shows pleiotropic effects in human cancer, and hence it has recently received increased attention of cancer researchers. After myriad investigations on HSF-1, the field has advanced to the phase where there is consensus that finding a potent and selective pharmacological inhibitor for this transcription factor will be a major break-through in the treatment of various human cancers. Presently, all reported inhibitors have their limitations, made evident at different stages of clinical trials. This brief account summarizes the advances with tested natural products as HSF-1 inhibitors and highlights the necessity of phytochemistry in this endeavor of discovering a potent pharmacological HSF-1 inhibitor.

Stress response, in plants and animals, produces bioactive compounds that offer persistent stress resistance and increases survival and longevity (*Xenohormesis*) (1). However, increasing survival is not always desired. A classic example of such a case is survival of cancer cells after treatment and recurrence of cancer with vigorous growth. Heat-shock factor-1 (HSF-1) is a primary translator of stress in eukaryotic cells. It is one of the well-characterized transcription factors in eukaryotic cells due to its comprehensive transcriptional activity to regulate many diseases (2, 3). Its fundamental role is to induce expression of heat-shock proteins (HSPs) in stressed cells, either due to heat stress or other cytotoxic stress, and assist the cells to repair the damage and survive. Even though this is a wonderful and extremely efficient natural process, which is conserved through evolution to protect organisms, this factor becomes detrimental in situations where intentional killing of cells is desired, such as treating cancer with both radiation and chemotherapy. Since HSF-1 is present both in healthy and malignant cells, it invariably protects the cells from cytotoxicity and the targeted DNA damage caused by chemotherapeutic agents or radiation therapy. Hence, the targeted cancer cells escape the death pathways and survive to relapse later as much more complicated drug-resistant cancer cells. Therefore, there is a need for a strategy to successfully inhibit HSF-1 in cancer cells. However, there is no such reliable inhibitor as a pharmacological drug on the market. This account briefly summarizes the development of the field of pharmacological inhibition of HSF-1 and the inevitable need of phytochemistry in discovering potential candidates, meeting all the requirements of an effective pharmaceutical, which could evolve as a adjunct therapeutic agent.

## HSF-1 Promotes Carcinogenesis and Tumor Growth

**Figure 1:**
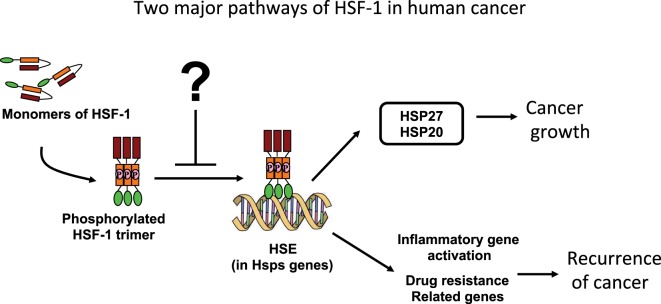
Schematic illustrations of pathways by which the heat-shock proteins (HSPs) interfere in human cancer prognosis.

The pathophysiology of carcinogenesis is regulated by many factors, primarily by the oncogenes. There are also many additional factors, which are not yet classified as oncogenes, that modulate the overall genesis and growth of cancer (4, 5). HSF-1 indeed has been reported to drive a transcriptional program in cancer cells, which is distinct from its conventional response to heat stress (6). Interestingly, key evidence has emerged in recent literature that HSF-1 is playing an important role in transforming benign tumors into malignant, and therefore converting cancer cell to “non-oncogenic addiction” (7). Moreover, many experimental animal models have revealed that HSF-1 indeed aggravated tumor xenograft growth. Carcinogenesis of skin cancer (4), promotion of hepatocellular carcinoma (8), and aggravation of breast cancer (9) are recent examples of how it is playing critical roles in cancer incidence, promotion, and invasion. Indeed, HSF-1 has been found to program a unique transcription scheme consisting of a spectrum of oncogenes (6). Also, human breast cancer tissue showed high abundance of HSF-1, and decreased level of HSF-1 has been linked to improved cancer prognosis (10). The knockout of HSF-1 impairs carcinogenesis and progression, suggesting that HSF-1 is a promising and potential therapeutic target in several cancers. There is a general consensus among scientists working in the field that discovering a potent and selective inhibitor of HSF-1 could be very valuable to suppress carcinogenesis and tumor growth (Figure 1).

## HSF-1 Inhibition Sensitizes Cancer Cell Death

Activation of HSF-1 is not only involved in carcinogenesis but it also enhances cancer cell survival post-chemotherapy or radiotherapy. For example, UV or doxorubicin-induced cancer cell death was found to be enhanced by inhibiting with HSF-1 (11, 12). HSF-1 is activated by phosphorylation through mitogen-activated protein kinases (MAPKs) (13, 14). Interestingly, both chemotherapeutic agents and radiation activate MAPKs. Hence, phosphorylation and activation of HSF-1 followed by protection of cancer cells occurs invariably during chemotherapy and radiation. After radiation or chemotherapy, HSPs are induced in cancer cells causing an anti-apoptotic effect (11, 12). Chemotherapy-induced HSPs trigger survival-signaling pathways, leading to relapse of cancer [e.g., GRP78 (15, 16) and Hsp27 (17, 18)]. Hence, HSF-1 promotes survival and proliferation of malignant cells.

It has been reported that the increased level of HSF-1 is associated with a reduced survival outcome in human breast and liver cancer (10, 19). In liver cancer, the increased expression of HSF-1 in peritumoral tissue is associated with early recurrence (20). A strong correlation was observed between HSF-1-positive tumor and worsened clinical outcome/mortality. The use of different approaches to inhibit HSF-1, alone or in combination with other target proteins, *in vitro* and in small animal models have shown promising results in increasing drug sensitivity and avoiding cancer relapse. Although there is no clinical investigation on inhibition of HSF-1 and demonstration of enhanced efficacy of cancer treatment, many preclinical studies, including our recent study (11), show promise for the potential to use as HSF-1 inhibitor as novel chemotherapeutic agents.

## Current Experimental Approaches of HSF-1 Inhibition

Naturally, pharmacological inhibition of transcription factors is very difficult to achieve due to a lack of potential target sites in their tertiary structures. HSF-1 is a ligand-less transcription factor with poor druggability. This is one of the reasons why achieving improved specificity for HSF-1 inhibition and experimental drugs is a difficult task. To overcome this, genetic approaches such as use of silencing RNAs are extensively pursued. Currently, both genetic and pharmacological approaches are being explored (21).

### Genetic Approaches

Small hairpin RNA (shRNA), which can target HSF-1 mRNA, sequence cloned expressions vectors have been successfully developed and demonstrated to knockdown HSF-1 in different cell types (21, 22). Typically, the shRNA target sequence containing sense and antisense oligonucleotides is cloned into an expression vector, and the cells are co-transfected with adeno- or lenti-viral DNA to generate target sequence containing viral particles. Both lenti- and adeno-virus based, viral titers have been used (23). Upon infection of the target cells with these viral particle, a hairpin RNA is produced, which is expected to bind to the HSF-1 mRNA preventing translation and decreasing total HSF-1 production. shRNA generation by lenti-viral vector was successfully employed recently to knockdown HSF-1 in human cancer cells (9). Another approach that was recently reported was to overexpress dominant-negative HSF-1 mutants. The dominant-negative HSF-1, which is usually appropriately engineered mutants, dilutes the transcriptional ability of endogenous HSF-1. These could be further modified to respond to a small molecular pharmaceutical (ligand regulated) either to activate (so that overall HSF1 transcriptional activity is inhibited) or to inhibit (in which case the endogenous HSF-1 activity is not affected) (24). However, serious limitations of these genetic approaches are that viral transduction methods are known to have poor efficacy and untoward side effects. In addition, FDA approval for retroviral agents for human use is complicated. Moreover, the World Health Organization has drafted a general restriction on the use of viral vector-based gene transfer or silencing for human application.

### Pharmacological Approaches

#### Direct Inhibition of HSF-1

**Figure 2:**
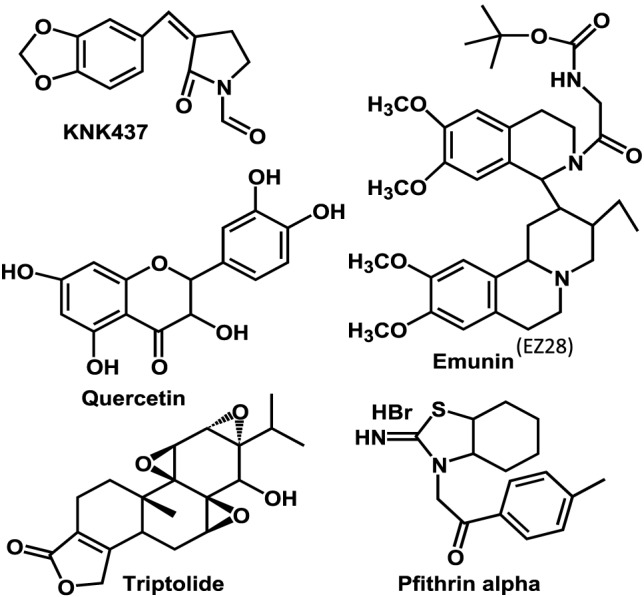
Molecular structures of heat-shock factor-1 (HSF-1) inhibitors currently evaluated.

Various pharmacological agents (Figure 2) have been developed to inhibit HSF-1 activity in various tissues and have been tested or are in clinical trial (3). The KNK 437 has been successfully demonstrated *in vitro* human cells that it could inhibit HSPs expression by inhibiting their transcription factor HSF-1. Triptolide, an active ingredient in the *Tripterygium wilfordii*, sometimes called *thunder god vine* but more properly translated *thunder duke vine*, was found to be a HSF-1 inhibitor (3). However, this compound could wide range of activity including immune suppression (25), and hence indicting the effects of Triptolide to the consequence of HSf-1 inhibition is not possible. Quercetin, a medicinally important member of the flavonoid family, is one of the most prominent dietary antioxidants. It is present in a variety of foods—including fruits, vegetables, tea, wine, as well as other dietary supplements—and is responsible for various health benefits (26). This has been shown to prevent the HSF-1 trimer binding to heat-shock elements in the DNA (3). Other drugs such as nucleotide analogs (Ly101-4B) were shown to inhibit HSF-1 expression in ovarian cancer cells (27). Overall, these drugs showed promises, and this approach is more viable and offers a more traditional introduction into the clinical realm. Each of these compounds has its complexity in use as a HSF-1 inhibitor (3). Pfithrin-α was originally developed as a p53 inhibitor but was later found to inhibit HSF-1 (28).

Other large quantity screening of compounds for potential inhibitors are undergoing. Recently an unbiased cell-based phenotypic screen was conducted to identify inhibitors of HSF-1. Among the 200,000 small molecules 4,6-disubstituted pyrimidine 1 with IC50 value of 2.00 µM was found to be good inhibitor (29). However, this class of compounds are also reported to be inhibiting CDK9 pathway (29), and therefore it is not clear whether the direct inhibition of HSF-1 or CDK2 is responsible for the inhibition of heat-shock response in the cells. Very recently, an elaborate attempt was made and reported the *de novo* development of a drug-like inhibitor hat targets human HSF-1 (30). In this approach, unlike any other previous attempts, potential drug-like inhibitor binding cavities in HSF-1 DNA binding domain (obtained from NMR and crystal structure of HSF-1/DNA complexes) was obtained to accommodate small drug-like molecules. Subsequently, a virtual screening of over 300,000 commercially available lead-like molecules were identified by identifying suitability for putative binding pockets and three-point pharmacophores. Then, 2,000 compounds were subjected to experimental screening and a potential candidate for HSF-1 inhibitor (I_HSF_, Fig) and its derivatives were proved to be inhibitor of HSF-1 transcriptional activity.

#### Inhibition of Secondary Targets

Heat-shock factor-1 is present in the cytoplasm as an inactive monomer, bound to other co-factors such as HSP90 (HSF-1/HSP90 complex). HSF-1 is released/untethered from the complex upon phosphorylation of Hsp90, and it becomes active by forming HSF-1 trimers. Therefore, inhibiting the phosphorylation of HSP90 could prevent the trimer formation. This approach has been attempted with novel pharmacological agents (31). Tanespimycin (17-*N*-allylamino-17-demethoxygeldanamycin, 17AAG) was developed with so much hope but before it was commercialized and clinical trials were stopped (32). HSF-1 functions both as an activator and a repressor of its target genes.

The approaches, used thus far, have been useful in determining mechanism but are not very clinically applicable. Unfortunately, drug(s) that selectively inhibit HSF-1 with high-binding activity have not been developed. Importantly, studies to date support efforts to identify and tailor novel drugs that specifically target HSF-1. There is an urgent need to explore natural product(s) for highly efficient and specific inhibition of HSF-1 and its transcriptional activity, or secondary targets. The use of pharmacological agents to inhibit HSF-1 is very clinically relevant, since the delivery of a single dose of a well-characterized and safe pharmacological agent is far more feasible and often safer than using genetic approaches.

## What the Future Holds for Phytochemists

Despite preclinical success of inhibiting HSF-1 in *in vitro* assays and successful demonstration of retardation of tumor growth in small animal models, each of the inhibitors listed above has limitations in translation into a drug for clinical use. As HSF-1 is important for both cancer and normal cells under stress conditions, systemic inhibition of HSF-1 could exert not only anticancer activity but also toxicity to normal cells. Therefore, for its use in cancer treatment, it is important to identify cancer cell specific inhibitors to minimize the cytotoxic effects on normal cells. This requires either fine-tuning of existing compounds by altering the functional groups/motifs by synthetic approaches or discovering and isolating new natural product(s) that can overcome the existing challenges. Such a potential pharmacological agent for HSF-1 inhibition could be identified from plants or plant-derived metabolites by screening a vast number of compounds. It is a potential opportunity for phytochemists to discover and isolate natural product(s) as a reliable inhibitor of HSF-1 to improve the lives of cancer patients. There are some compounds that have been identified as potential leads. A terpenoid, cantharidin (33), isolated from blister beetles and other insects, induces cancer cell death by blocking HSF-1 binding to target gene promoters and subsequently inhibits transcription. Also, in cancer cells, the protein translation initiation inhibitor rocaglates, secondary metabolites of plant genus *Aglaia*, inactivates the transcriptional activity of HSF-1 (34). A serious limitation with all these agents is their lack of specificity, cellular toxicity, and deleterious effects on normal cells. Targeting Hsp27, by natural products and plant extracts, is also found to be very effective way of killing cancer cells (35, 36).

There are also multiple evidences that stress can produce bioactive compounds in plants, and these compounds have been proven to offer anti-carcinogenic effect in small animal models and human, and therefore to be useful in cancer therapy as dietary supplements. Examples of such compounds are increase of curcumin content in stressed *Curcuma longa*, increase of Jasmonate in stressed *Arabidopsis* (Mustard), higher Carvacrol in stressed *Oregano x majoricum*, and so on [refer the Table 1 in the reference (1)]. It is not currently known how the HSF-1 is involved in the increased production of anticancer agents in these stressed plants, and whether inhibiting HSF-1 activity in these plants will reduce the contents of these cancer-inhibiting compounds. Also, dietary supplementation of the stress-inducible natural products is proven to be effective in treating cancer. However, it is not well established that the beneficial effect of these compounds are due to inhibition of HSF-1 in cancer cells. As these compounds associate with multiple targets, it is extremely difficult to determine whether HSF-1 inhibition is a major contributor to the observed positive effect. Only a few compounds have been thoroughly studied for their action on HSF-1 in cancer cells. For example, resveratrol, a phenolic compound, found in grape seed, inhibits Akt phosphorylation to suppress HSF-1 activation in cancer cells, so that the efficacy is increased (37). Contrarily, Curcumin, an established anticancer agent, increases HSP expression, likely due to activation of HSF-1 (38). This paradox further strengthens our perception that highly specific HSF-1 inhibitors are essential, not only to use as a cancer therapeutic but it is also required to understand HSF-1-dependent mechanistic pathways of these nutraceuticals in laboratory studies. Therefore, identifying a suitable inhibitor for successful inhibition of HSF-1 to assist/enhance treatment outcome of human cancer should come from the hands of phytochemists, who can isolate the natural compounds and modify the efficacy of these inhibitors.

## Author Contributions

All authors contributed intellectually and in writing the manuscript.

## Conflict of Interest Statement

The authors declare that the research was conducted in the absence of any commercial or financial relationships that could be construed as a potential conflict of interest.
